# Association of the insulin resistance marker TyG index with the severity and mortality of COVID-19

**DOI:** 10.1186/s12933-020-01035-2

**Published:** 2020-05-11

**Authors:** Huihui Ren, Yan Yang, Fen Wang, Yongli Yan, Xiaoli Shi, Kun Dong, Xuefeng Yu, Shujun Zhang

**Affiliations:** Department of Endocrinology, Tongji Hospital, Tongji Medical College, Huazhong University of Science and Technology, Wuhan, 430030 Hubei People’s Republic of China

**Keywords:** TyG index, COVID-19, Mortality, Severity

## Abstract

**Background:**

The triglyceride and glucose index (TyG) has been proposed as a marker of insulin resistance. This study aims to evaluate the association of the TyG index with the severity and mortality of coronavirus disease 2019 (COVID-19).

**Methods:**

The study included a cohort of 151 patients with COVID-19 admitted in a tertiary teaching hospital in Wuhan. Regression models were used to investigate the association between TyG with severity and mortality of COVID-19.

**Results:**

In this cohort, 39 (25.8%) patients had diabetes, 62 (41.1%) patients were severe cases, while 33 (22.0%) patients died in hospital. The TyG index levels were significantly higher in the severe cases and death group (mild vs. severe 8.7 ± 0.6 vs. 9.2 ± 0.6, *P *< 0.001; survivor vs. deceased 8.8 ± 0.6 vs. 9.3 ± 0.7, *P *< 0.001), respectively. The TyG index was significantly associated with an increased risk of severe case and mortality, after controlling for potential confounders (OR for severe case, 2.9, 95% CI 1.2–6.3, *P *= 0.007; OR for mortality, 2.9, 95% CI 1.2–6.7, *P *= 0.016). The associations were not statistically significant for further adjustment of inflammatory factors.

**Conclusion:**

TyG index was closely associated with the severity and morbidity in COVID-19 patients, thus it may be a valuable marker for identifying poor outcome of COVID-19.

## Background

In December 2019, an outbreak of pneumonia caused by the 2019 novel coronavirus disease was first observed in Wuhan, China [1]. On Feb 11 2020, the World Health Organization (WHO) officially named the disease as Coronavirus Disease 2019 (COVID-19). By Feb 19, 2020, a total of 74 283 cases with COVID-19 in China have been reported, with 2009 (2.7%) death [2]. Although most patients with COVID-19 have mild symptoms and good prognosis [3], accumulating evidence have indicated a high mortality of the COVID-19 patients (11%) [1, 4], resembling that of severe acute respiratory syndrome coronavirus (SARS-CoV) infection [5]. A few patients rapidly developed severe pneumonia, acute respiratory distress syndrome, cardiac injury, or multiple organ failure and died [4]. However, data on the clinical characteristics of patients with COVID-19 are limited, and the underlying mechanism is not clear yet. Therefore, early identification of the severity of COVID-19 is very important for public health.

Recently, the triglyceride-glucose (TyG) index, a product of triglycerides and glucose, is widely used as a reliable surrogate marker of insulin resistance (IR) [6]. Studies have shown that the TyG index is associated with an increased risk of diabetes [7], hypertension [8], nonalcoholic fatty liver disease [9], and might predict the development of cardiovascular events [10]. Patients with cardiometabolic disorders may be more susceptible to infection of COVID-19 and becoming severe case, partly due to their abnormal metabolism and systemic inflammation state. Thus, these patients might have a higher risk of poor outcome. In this sense, studies that evaluate the relationship between TyG index with the severity and mortality of COVID-19 are urgently needed.

In this study, we investigated patients with confirmed COVID-19 who were admitted to Wuhan Tongji hospital. The purpose of this study was to explore the association of TyG index levels with the clinical outcomes of patients with COVID-19 and to provide ideas for improving COVID-19 prognosis.

## Methods

### Study population

This study retrospectively analysed confirmed COVID-19 from Jan 12 2020, to Feb 13 2020 at Tongji Hospital of Huazhong University of Science and Technology in Wuhan, China. All patients enrolled in this study were diagnosed as COVID-19 according to WHO interim guidance [11]. Patients with missing data on clinical characteristics were excluded. Considering the complexity of the disease, as well as the uncertainty of the course of treatment, 151 patients which had completed medical records and follow up data were included. This study was approved by the Ethics Commission of Tongji Hospital, and all participants provided oral informed consent.

### Data collection

We reviewed the epidemiological, demographic, clinical, and laboratory data from patients’ medical records. Two researchers checked the data collected independently. We collected information on socioeconomic characteristics (age, sex, exposure history), smoking, chronic medical histories (diabetes, hypertension, cardiovascular disease, cerebrovascular disease, chronic kidney disease, chronic pulmonary disease, chronic liver disease), clinical symptoms (fever, cough, expectoration, dyspnoea, chest pain, diarrhea, headache, hypodynamia, anorexia, nausea and vomiting), vital signs (temperature, heart rate, respiratory rate, oxygen saturation, blood pressure), laboratory data (complete blood count, liver and renal function, fasting plasma glucose (FPG), hemoglobin A1c (HbA1c), lipid, high-sensitivity C-reactive protein (CRP), Ferritin, and tumor necrosis factor α (TNFα)), as well as living status during study period.

### Definitions

The TyG index was calculated by the formula ln [fasting triglycerides (mg/dL) × FPG (mg/dL)/2]. The patients were divided into 3 tertiles according to TyG index levels, T1 (n = 50, 7.5 ≤ TyG index ≤ 8.6), T2 (n = 51, 8.7 ≤ TyG index ≤ 9.1), T3 (n = 50, 9.2 ≤ TyG index ≤ 10.7). Diabetes was defined as self-reported medical history of diabetes, and the use of antidiabetic drugs. Hypertension was defined as self-reported history of hypertension, and the use of antihypertensive drugs. Other chronic diseases, such as cardiovascular disease, cerebrovascular disease, chronic kidney disease, chronic pulmonary disease, and chronic liver disease, were diagnosed according to self-reported medical history.

Severity of the disease was staged according to the guidelines for diagnosis and treatment of COVID-19 (trial sixth edition) published by National Health Commission of China on February 18, 2020. Severe case was defined as including one criterion as follow: 1. respiratory rate > 30/min, 2. oxygen saturation ≤ 93%, 3. PaO2/FiO2, 4. Patients developed either with shock, or respiratory failure requiring mechanical ventilation, or combined with the other organ failure admission to intensive care unit (ICU).

### Statistical analysis

We presented continuous variables as mean ± standard deviation (SD) for normally distributed data or median (interquartile range, IQR) for data with skew distribution, and categorical variables as frequencies (percentage,  %). We assessed differences between mild and severe cases using two-sample *T* test or Mann–Whitney *U* test depending on parametric or nonparametric data for continuous variables and Chi square test for categorical variables. Comparisons among TyG index groups were assessed by the analysis of variance (ANOVA) or Kruskal–Wallis test. Univariate and multivariate logistic regression analyses were used to evaluate the associations between TyG index and the severity and mortality of COVID-19. The associations of TyG index with severity of COVID-19 in subgroups classified by age (< 60 vs. ≥ 60 years), sex (male vs. female), and medical history of chronic disease (presence vs. absence), including diabetes mellitus and hypertension, were also studied using logistic regression analysis. We used SPSS software, version 19.0 (SPSS, Inc., Chicago, IL, USA) for all analyses with statistically significant for *P* < 0.05.

## Results

A total of 151 admitted hospital patients were included in our study, with a mean age of 59.5 ± 15.9 years old. 78 (51.7%) of patients were males. Regarding the history of chronic diseases, 39.7% had hypertension, 25.8% had diabetes, 10.6% had cardiovascular disease, 4.6% had cerebrovascular disease, 7.3% had chronic kidney disease, 1.3% had chronic pulmonary disease, and 2.0% had chronic liver disease. The most prevalent symptoms were fever (88.7%), cough (68.8%), and fatigue (56.3%). In this population, 62 (41.1%) patients were severe cases, while 33 (22.0%) patients died in hospital. The baseline characteristics of patients according to severity were shown in Table 1. Severe patients were older, more males, and more likely to have hypertension and diabetes compared with mild patients. The prevalence of dyspnea, anorexia, and fatigue of the severe group were higher than that of the mild group (all *P* < 0.05). Moreover, severe patients had significantly higher value of respiratory rate, systemic blood pressure (SBP), and mortality and/or more likely to receive auxiliary ventilation, and invasive mechanical ventilation (all *P *< 0.05), but there was no markedly difference for other parameters.

**Table 1:** The basic clinical features of the COVID-19 patients according to the severity

	Total (N = 151)	Mild cases (N = 89)	Severe cases N = 62)	P
Age, mean ± SD, years	59.5 ± 15.9	53.9 ± 16.2	67.6 ± 11.6	0.000
Sex, No. (%)				
Male	78 (51.7%)	38 (42.7%)	40 (64.5%)	0.008
Female	73 (48.3%)	51 (57.3%)	22 (35.5%)	
Comorbidities, No. (%)				
Diabetes	39 (25.8%)	16 (18.0%)	23 (37.1%)	0.008
Hypertension	60 (39.7%)	25 (28.1%)	35 (56.5%)	0.000
Cardiovascular disease	16 (10.6%)	7 (7.9%)	9 (14.5%)	0.191
Cerebrovascular disease	7 (4.6%)	2 (2.2%)	5 (8.1%)	0.094
Chronic kidney disease	11 (7.3%)	5 (5.6%)	6 (9.7%)	0.345
Chronic pulmonary disease	2 (1.3%)	1 (1.1%)	1 (1.6%)	0.796
Chronic liver disease	3 (2.0%)	2 (2.2%)	1 (1.6%)	0.783
Signs and symptoms				
Fever, No. (%)	134 (88.7%)	79 (88.8%)	55 (88.7%)	0.992
Cough, No. (%)	104 (68.9%)	58 (65.2%)	46 (74.2%)	0.239
Expectoration, No. (%)	61 (40.4%)	37 (41.6%)	24 (38.7%)	0.724
Dyspnea, No. (%)	78 (51.7%)	38 (42.7%)	40 (64.5%)	0.008
Pectoralgia, No. (%)	11 (7.3%)	7 (7.9%)	4 (6.5%)	0.742
Diarrhoea, No. (%)	54 (35.8%)	34 (38.2%)	20 (32.3%)	0.453
Nausea, No. (%)	24 (15.9%)	16 (18.0%)	8 (12.9%)	0.401
Vomiting, No. (%)	10 (6.6%)	6 (6.7%)	4 (6.5%)	0.944
Anorexia, No. (%)	57 (37.7%)	27 (30.3%)	30 (48.4%)	0.024
Headache, No. (%)	28 (18.5%)	14 (15.7%)	14 (22.6%)	0.287
Fatigue, No. (%)	85 (56.3%)	44 (49.4%)	41 (66.1%)	0.042
Tmax, mean ± SD, °C	38.5 ± 0.7	38.5 ± 0.7	38.7 ± 0.7	0.148
Respiratory rate, mean ± SD, bpm	23 ± 5	21 ± 4	26 ± 6	0.000
Heart rate, mean ± SD, bpm	92 ± 18	90 ± 16	94 ± 20	0.172
Systolic blood pressure, mean ± SD, mmHg	133 ± 20	129 ± 18	138 ± 20	0.007
Diastolic blood pressure, mean ± SD, mmHg	82 ± 13	82 ± 12	81 ± 14	0.531
Auxiliary ventilation, No. (%)	46 (30.5%)	0	46 (74.2%)	0.000
Invasive mechanical ventilation, No. (%)	15 (9.9%)	0	15 (24.2%)	0.000
Mortality, No. (%)	33 (22.0%)	0	33 (54.1%)	0.000

*SD* standard deviation, *Tmax* maximum body temperature

P values indicate differences between mild and severe cases. P < 0.05 was considered statistically significant

Laboratory findings of patients according to the severity were shown in Table 2. For blood routine examination, severe patients had obviously higher values of white blood cell (WBC) count and neutrophil count (NEU), but notably lower levels of lymphocytes count than mild patients (*P *< 0.05). Regarding blood biochemical indicators, severe group had higher levels of aspartate aminotransferase (AST), lactate dehydrogenase (LDH), creatinine, uric acid (UA), CRP, and FPG, compared with mild group (*P *< 0.05). In addition, the levels of Ferritin and TNFα were also elevated in severe ones (*P *< 0.05). Notably, TyG index, which calculated by triglycerides and glucose, was markedly higher in severe cases than in mild ones (*P *< 0.05).

**Table 2:** Laboratory findings of patients with COVID-19 on admission to hospital

	Normal range	Mild cases (N = 89)	Severe cases (N = 62)	P
White blood cell count, × 10^9^/L	3.5–9.5	4.9 (2.0)	7.3 (6.7)	0.000
Neutrophil count, × 10^9^/L	1.8–6.3	3.0 (2.1)	5.8 (7.1)	0.000
Lymphocyte count, × 10^9^/L	1.1–3.2	1.2 ± 0.5	0.8 ± 0.4	0.000
Alanine aminotransferase, U/L	≤ 33	28 ± 32	34 ± 27	0.266
Aspartate aminotransferase, U/L	≤ 32	24.0 (13.0)	36.0 (26.0)	0.000
Lactate dehydrogenase, U/L	135–214	251.0 (93.0)	431.5 (367.0)	0.000
Creatinine, μmol/L	45–84	70.0 (28.5)	81.5 (33.0)	0.003
Uric acid, μmol/L	202.3–416.6	248.0 (103.9)	264.5 (168.9)	0.036
Total cholesterol, mmol/L	< 5.18	3.8 ± 0.7	3.6 ± 0.8	0.089
C-reactive protein, mg/L	< 1	12.6 (36.4)	68.7 (114.6)	0.000
Erythrocyte sedimentation rate, mm/H	0–20	28 ± 22	32 ± 22	0.477
Ferritin, ug/L	15–150	888.2 ± 2181.7	1869.1 ± 2229.4	0.027
TNFα, pg/mL	< 8.1	7.3 (3.3)	9.1 (6.0)	0.002
HbA1c, %	4–6	6.0 ± 1.5	6.7 ± 2.1	0.083
Fasting glucose, mmol/L	4.11–6.55	6.2 (2.3)	8.4 (5.8)	0.000
Triglyceride, mmol/L	< 1.7	1.4 ± 1.0	1.5 ± 0.6	0.431
TyG	–	8.7 ± 0.6	9.2 ± 0.6	0.000

Continuous data were expressed as mean ± SD or median (interquartile range)

P values indicate differences between mild and severe cases. P < 0.05 was considered statistically significant

**Fig. 1:**
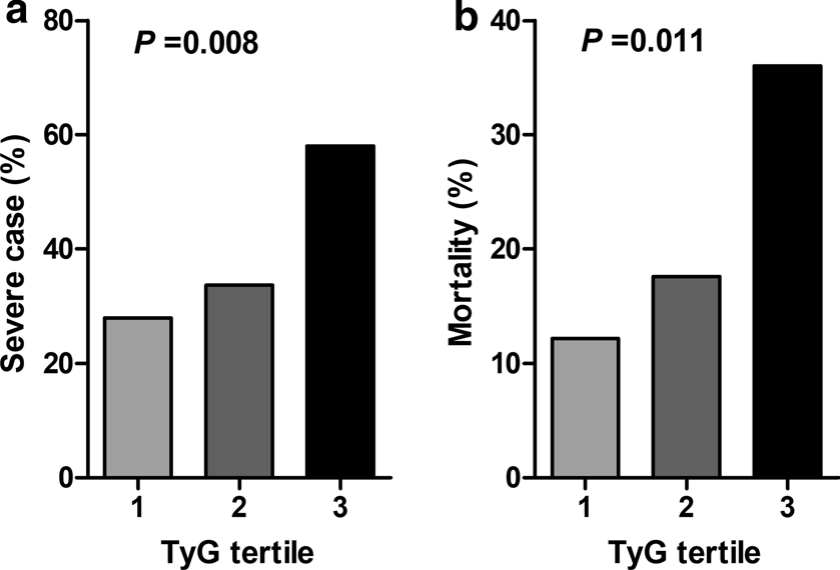
Frequency of severe cases **a** and death **b** of COVID-19 patients according to the tertiles of TyG index

Table 3 showed the baseline characteristics among COVID-19 patients, divided according to TyG index tertiles. Compared to the first tertile of TyG index, patients in the highest tertile of TyG presented higher levels of WBC, NEU, AST, LDH, CRP, and ferritin, yet lower levels of lymphocytes count (all *P* < 0.05). Most importantly, patients with increasing level of TyG index had a higher incidence of severe COVID-19 case and death (*P* = 0.008 and 0.01, respectively), as shown in Fig. 1.

**Table 3:** Characteristics of the COVID-19 patients according to the tertiles of TyG

	TyG tertiles			P
	T1 (7.5–8.6) (N = 50)	T2 (8.7–9.1) (N = 51)	T3 (9.2–10.7) (N = 50)	
Age, years	53 (32)	66 (22)	67.5 (18)	0.006
Male, No. (%)	21 (42%)	25 (49%)	32 (64%)	0.080
White blood Cell count, × 10^9^/L	4.5 (1.9)	5.5 (4.0)	6.5 (4.7)	0.000
Neutrophil count, × 10^9^/L	2.7 (1.5)	4.0 (4.6)	4.6 (5.0)	0.000
Lymphocyte count, × 10^9^/L	1.2 ± 0.5	1.0 ± 0.6	0.9 ± 0.6	0.018
Aspartate aminotransferase, U/L	23 (18)	29 (13)	33.5 (29)	0.016
Lactate dehydrogenase, U/L	240.0 (130.0)	293.0 (176.0)	332.0 (245.0)	0.000
Creatinine, μmol/L	73.1 ± 27.6	78.8 ± 31.6	86.9 ± 34.3	0.089
Uric acid, μmol/L	260.2 ± 80.6	266.3 ± 122.4	287.2 ± 129.2	0.455
C-reactive protein, mg/L	11.6 (37.9)	39.8 (70.3)	37.9 (127.6)	0.005
Ferritin, ug/L	377.8 (470.4)	642.7 (1247.2)	1008.5 (1154.1)	0.000
TNFα, pg/mL	7.4 (2.2)	8.8 (5.5)	9.4 (6.3)	0.012
Fasting glucose, mmol/L	5.5 (1.2)	6.5 (2.1)	10.8 (7.8)	0.000
Triglyceride, mmol/L	0.9 (0.3)	1.4 (0.5)	1.8 (1.0)	0.000
TyG	8.3 (0.4)	8.9 (0.3)	9.6 (0.6)	0.000

Continuous data were expressed as mean ± SD or median (interquartile range)

P values indicate differences among the tertiles of TyG. P < 0.05 was considered statistically significant

Additionally, the association between TyG index and the severity of COVID-19 were further confirmed by logistic regression (Table 4). After adjustment for age, sex, SBP and HbA1C, the TyG index was independently associated with the severity of COVID-19 patients with an odds ratio (OR) of 2.9 [95% confidence interval (CI) 1.26–6.3]. However, no significant association was observed after further adjusting for CRP and TNFα (OR 2.3, 95% CI 0.6–8.3).

**Table 4:** Odds ratios for severe cases associated with TyG in whole population and in different subgroups

	Model1		Model2		Model3	
	OR (95% CI)	P	OR (95% CI)	P	OR (95% CI)	P
Total	2.8 (1.6–4.8)	0.000	2.9 (1.2–6.3)	0.007	2.3 (0.6–8.3)	0.207
Subgroups						
Sex						
Male	3.6 (1.6–8.2)	0.002	5.7 (1.7–19.2)	0.005	3.2 (0.4–22.7)	0.246
Female	2.8 (1.6–4.8)	0.000	3.3 (1.5–7.0)	0.003	2.6 (0.8–9.0)	0.132
Age						
≥ 60 years	2.6 (1.2–5.7)	0.013	3.0 (1.2–7.3)	0.014	5.4 (1.0–30.6)	0.055
< 60 years	1.8 (0.7–4.9)	0.246	1.3 (0.2–8.5)	0.773	0.6 (0.1–9.7)	0.746
DM						
Yes	6.5 (1.4–29.6)	0.016	6.8 (1.3–34.8)	0.021	6.0 (0.7–51.7)	0.103
No	1.9 (0.9–3.7)	0.077	1.2 (0.6–2.7)	0.630	1.2 (0.4–4.0)	0.780
Hypertension						
Yes	4.6 (1.6–13.3)	0.006	4.3 (1.0–18.7)	0.054	6.3 (0.6–64.2)	0.121
No	1.7 (0.8–3.5)	0.138	1.8 (0.6–5.3)	0.269	1.7 (0.3–8.6)	0.520

Model1: unadjusted

Model2: adjusted for age, sex, HbA1C, and SBP

Model3: adjusted for age, sex, SBP, HbA1C, CRP, and TNFα

*OR* odds ratio, *CI* confidence interval, *DM* diabetes mellitus, *SBP* systolic blood pressure, *CRP* C-reactive protein

Next, we studied correlations between TyG with CRP and TNFα by using Pearson correlation analysis. We observed that after controlling for age, sex, HbA1c, and SBP, TyG levels positively correlated with both CRP (*r *= 0.286, *P *= 0.003) and TNFα (*r *= 0.42, *P *< 0.0001).

We further assessed the effect of TyG index in particular subsets of patients with COVID-19 (Table 4). Similar results were observed in subgroups analysis. After adjustment for age, sex, SBP and HbA1C, the positively association between TyG index and the severity was observed in subgroups of men, women, age ≥ 60, and patients with diabetes (OR 5.7, 95% CI 1.7–19.2; OR 3.3, 95% CI 1.5–7.0; OR 3.0, 95% CI 1.2–7.3; OR 6.8, 95% CI 1.3–34.8). However, the adjusted association was not statistically significant observed after adding CRP and TNFα in model 3 (OR 3.2, 95% CI 0.4–22.7; OR 2.6, 95% CI 0.8–9.0; OR 5.4, 95% CI 51.0–30.6; OR 6.0, 95% CI 0.7–51.7). Additionally, the associations were not significant in patients with age < 60 years, or without diabetes or hypertension.

Moreover, the results showed the TyG index level was significantly higher in the death group (survivor vs. deceased 8.8 ± 0.6 vs. 9.3 ± 0.7, *P *< 0.001). We used a logistic regression model to evaluate the association of TyG index with the mortality of COVID-19, and found that the TyG index was a risk factor for increased mortality of COVID-19 patients (OR 2.9, 95% CI 1.2–6.7) after adjustment for age, sex, SBP and HbA1C (Table 5). Notably, the association was not statistically significant observed after additionally adjustment for CRP and TNFα (OR 2.8, 95% CI 0.7–11.3).

**Table 5:** Association of TyG and mortality in total population

	Survival	Death	P
TyG levels	8.8 ± 0.6	9.3 ± 0.7	0.000
OR (95% CI)			
Model1	1	3.0 (1.6–5.6)	0.001
Model2	1	2.9 (1.2–6.7)	0.016
Model3	1	2.8 (0.7–11.3)	0.153

Model1: unadjusted

Model2: adjusted for age, sex, SBP and HbA1C

Model3: adjusted for age, sex, SBP, HbA1C, CRP, and TNFα

*OR* odds ratio, *CI* confidence interval, *SBP* systolic blood pressure, *CRP* C-reactive protein

Finally, we performed the receiver operating characteristic (ROC) curve analysis to test the ability of TyG to predict the severity and mortality of COVID-19. The optimal cut-off value of TyG was derived from the point with the maximum Youden index. The area under the ROC curve (AUROC) of TyG for predicting severe COVID-19 patients was 0.664 (95% CI 0.576–0.751) (*P *= 0.001). The optimal cut-off point of TyG was 8.5, with a sensitivity of 87.1% and specificity of 38.2%. Moreover, the AUROC of TyG for predicting the mortality of COVID-19 was 0.687 (95%CI 0.584–0.790) (*P *= 0.001). The optimal cut-off point of TyG was 9.6, yielding sensitivity and specificity of 39.4% and 92.3%, respectively.

## Discussion

In this study, we evaluated the role of TyG index in identifying severe cases and mortality of COVID-19 patients. We demonstrated for the first time that the incidence of severe COVID-19 case was higher among patients with increasing level of TyG index. After adjusting confounding factors, the TyG index was closely associated with an increased risk of severe patients with COVID-19. In addition, TyG index was also a valuable predictor for mortality of COVID-19.

### TyG index and the severity and mortality of COVID-19

Epidemiological evidence have shown a high mortality and morbidity of diabetic patients who develop COVID-19 [4]. Our data also observed a high prevalence of diabetes in COVID-19 patients. Notably, we found that the TyG index, a reliable surrogate marker of IR, were predictive of severe cases and mortality of COVID-19 patients. Previous studies have shown that the TyG index may predict the development of type 2 diabetes mellitus [12] and metabolic syndrome [13]. It has been reported that the TyG index is positively associated with the higher prevalence of subclinical coronary artery disease (CAD) [14], symptomatic CAD and metabolic and behavioral risk factors [15], and arterial stiffness and nephric microvascular damage [16]. Moreover, higher TyG index correlated with increased risk of main adverse cardiovascular and cerebrovascular events (MACCE) in acute ST-elevation myocardial infarction (STEMI) patients, and the TyG index might be a valuable predictor of clinical outcomes in STEMI patients undergoing percutaneous coronary intervention [17]. Moreover, an elevated TyG index was significantly associated with a higher risk of arterial stiffness and nephric microvascular damage. The positive association of TyG index with increased risk of developing severe cases and the risk of mortality in confirmed COVID-19 patients, could be attributed to the roles of IR in metabolic disorder, an altered inflammation profile, and cardiovascular disease risk factors. These findings support the significance of TyG index as a valuable predictor of poorer outcomes for COVID-19 patients, and remind physicians that more intensive attention should be paid to patients with high level of TyG index.

### Inflammation and the severity and mortality of COVID-19

The virus of COVID-19 was classified as beta-coronavirus, and had highly pathogenicity similar to SARS-CoV and middle east respiratory syndrome coronavirus (MERS-CoV), that could cause severe respiratory syndrome in humans [18–20]. Yet, the fundamental pathophysiology of COVID-19 remains unknown. Previous studies have shown that increased concentrations of proinflammatory cytokines in serum (e.g., IL1B, IL6, IL12, IFNγ, IL10, and TNFα) were induced in patients with SARS [21] and MERS [22]. Considering the large amount of cytokines induced by SARS-CoV and MERS-CoV, a cytokine storm in the body might play a crucial role in pulmonary inflammation and extensive lung damage of patients with COVID-19. In early studies, COVID-19 was also reported to induce increased secretion of T-helper-2 (Th2) cytokines (e.g., IL4 and IL10) that suppress inflammation [4]. We noted that severe patients of COVID-19 had high amounts of inflammatory indicators (Ferritin and TNFα) in severe patients than mild ones, suggesting that the cytokine storm was associated with disease severity.

### Other potential factors and the severity and mortality of COVID-19

Consistent with previous reported, older patients were more susceptible to COVID-19, and 70% of infected patients were men [1]. In the present study, similar results were observed in age, yet there is no difference in the prevalence of male or female in total patients with COVID-19. Notably, the present study also emphasized a greater number of men among severe cases compared with mild cases. These findings suggest that older, male patients was more likely to have a worse prognosis. In addition to the elderly and males, we found that patients with a history of diabetes and hypertension are at increased risk of severe COVID-19 and mortality, in agreement with previous studies [4, 23]. Nevertheless, we observed a higher prevalence of diabetes and hypertension than that reported in the above study. The high prevalence of comorbid conditions may be expected for Tongji Hospital being the designated hospital for severe cases.

We found TyG index was closely associated with the mortality of COVID-19, which may be useful to predict the poor outcome for the infected patients. Although we attempted to adjust for many confounders, it is difficult to assess the independently cause of death among the 33 dead cases. The causes of death may be respiratory failure, heart failure, inflammation storm, sepsis, disseminated intravascular coagulation, multiple organ failure, acute kidney injury, malignant arrhythmia, diabetic ketoacidosis, even sudden death, and other uncertain events. Taken together, it is not easy to identify the relationship between TyG index and any cause of death in the world.

### Study strengths and limitations

This study has several strengths. First, the COVID-19 was first observed in Wuhan China. A large number of patients are suffering with COVID-19 in this city, which provided us an opportunity to evaluate the epidemic affair. Second, we benefited from obtaining accurate and thorough clinical data of patients in Tongji Hospital, which is a designated hospital responsible for the treatments for moderate-severe COVID-19 patients in Wuhan. Third, all authors are first line doctors, who could directly observe participants.

Several limitations in this study should also be addressed. First, considering the complexity of the disease, as well as the limited information on the course of disease, the number of samples is relatively small. Second, compared with published paper, the percentage of severe cases was high in our study. It may be explained by that Tongji Hospital is the designated hospital for moderate-severe cases. Third, most people were hospitalized for treatment of pneumonia, resulting that metabolic disorders were easily overlooked. Thus, we could not observe the association of metabolic control with the prognosis of COVID-19. Fourth, the TyG index, which predicted the death of COVID-19 in this study, might be affected by the change of triglycerides and glucose levels. Other factors, such as inflammation, should also be taken into consideration when predicting the death of a patient.

## Conclusion

In summary, our study illustrated that the TyG index was notably high in severe and dead patients of COVID-19. Moreover, the study unveiled that TyG index was an independent predictor of an increased risk of poor outcome in patients with COVID-19. These findings also highlighted the high risk of severe condition and death among patients with metabolic disorders. Despite this, understanding the mechanisms underlying the interaction of the TyG index and COVID-19 will allow for drug development aimed at treatment and/or preventing severe condition.

## Data Availability

The datasets generated and analysed for this study are available from the corresponding author upon reasonable request.

## Abbreviations

COVID-19: Corona virus disease 2019
SARS-CoV: Severe acute respiratory syndrome coronavirus
TyG: The triglyceride-glucose index
IR: Insulin resistance
FPG: Fasting plasma glucose
HbA1c: Hemoglobin A1c
CRP: C-reactive protein
TNFα: Tumor necrosis factor α
ICU: Intensive care unit
SD: Standard deviation
IQR: Interquartile range
ANOVA: Analysis of variance
SBP: Systemic blood pressure
WBC: White blood cell
NEU: Neutrophil count
AST: Aspartate aminotransferase
LDH: Lactate dehydrogenase
UA: Uric acid
OR: Odds ratio
CI: Confidence interval
ROC: Receiver operating characteristic curve
AUROC: Area under the receiver operating characteristic curve
CAD: Coronary artery disease
MACCE: Main adverse cardiovascular and cerebrovascular events
STEMI: ST-elevation myocardial infarction
MERS-CoV: Middle east respiratory syndrome coronavirus
